# Optimizing the treatment of metastatic castration-resistant prostate cancer: a Latin America perspective

**DOI:** 10.1007/s12032-018-1105-8

**Published:** 2018-03-19

**Authors:** Juan Pablo Sade, Carlos Alberto Vargas Báez, Martin Greco, Carlos Humberto Martínez, Miguel Ángel Álvarez Avitia, Carlos Palazzo, Narciso Hernández Toriz, Patricia Isabel Bernal Trujillo, Diogo Assed Bastos, Fabio Augusto Schutz, Santiago Bella, Lucas Nogueira, Neal D. Shore

**Affiliations:** 1 Instituto Alexander Fleming, Buenos Aires, Argentina; 2 Universitario Fundacion Santa Fe de Bogota, Bogota, Colombia; 3 Centro de Educación Médica e Investigaciones Clínicas, Buenos Aires, Argentina; 4 Unidad de Cancerología, Departamento de Cirugía, División de Urología, Hospital Pablo Tobón Uribe Medellín, Antioquia, Colombia; 5 Instituto Nacional de Cancerologia, Mexico City, Mexico; 6 Department of Uro-Oncology, Instituto de Diagnóstico y Tratamiento Sagrada Familia, Tucumán, Argentina; 7 Hospital de Oncología Centro Médico Nacional Siglo XXI, Mexico City, Mexico; 8 Department of Nuclear Medicine, Fundación Santa Fe de Bogota, Bogota, Colombia; 9 Hospital Sírio- Libanês and Uro-Oncology Department, Instituto do Câncer do Estado de São Paulo (ICESP), São Paulo, Brazil; 10 Hospital São José, São Paulo, Brazil; 11 Universidad Católica de Córdoba and the Clínica Universitaria Reina Fabiola, Córdoba, Argentina; 12 MD Hospital das Clínicas, Universidade Federal de Minas Gerais, Belo Horizonte, Brazil; 13 Department of Urology, Carolina Urologic Research Center, 823 82nd Parkway, Myrtle Beach, SC 29572 USA

**Keywords:** Chemotherapy, Hormonal therapy, Radium-223, Latin America, mCRPC

## Abstract

Prostate cancer is a significant burden and cause of mortality in Latin America. This article reviews the treatment options for patients with metastatic castration-resistant prostate cancer (mCRPC) and provides consensus recommendations to assist Latin American prostate cancer specialists with clinical decision making. A multidisciplinary expert panel from Latin America reviewed the available data and their individual experience to develop clinical consensus opinions for the use of life-prolonging agents in mCRPC, with consideration given to factors influencing patient selection and treatment monitoring. There is a lack of level 1 evidence for the best treatment sequence or combinations in mCRPC. In this context, consensus recommendations were provided for the use of taxane-based chemotherapies, androgen receptor axis-targeted agents and targeted alpha therapy, for patients in Latin America. Prostate-specific antigen (PSA) changes alone, during treatment, should be treated with caution; PSA may not be a suitable biomarker for radium-223. Bone scans and computed tomography are the standard imaging modalities in Latin America. Imaging should be prompted during treatment where symptomatic decline and/or significant worsening of laboratory evaluations are reported, or where a course of therapy has been completed and another antineoplastic agent is under consideration. Recommendations and guidance for treatment options in Latin America are provided in the context of country-level variable access to approved agents and technologies for treatment monitoring. Patients should be treated with the purpose of prolonging overall survival and preserving quality of life through increasing the opportunity to administer all available life-prolonging therapies when appropriate.

**Electronic supplementary material:**

The online version of this article (10.1007/s12032-018-1105-8) contains supplementary material, which is available to authorized users.

## Introduction

Worldwide, prostate cancer is the second most commonly diagnosed cancer in men accounting for 7% of male cancer deaths [1]. In 2012, approximately 134,000 new prostate cancer cases and 43,000 deaths from prostate cancer were estimated in Central and South America, accounting for 12% of all cancer cases and 14% of male cancer deaths. Across this region, prostate cancer was the most common male cancer diagnosis and one of the leading causes of cancer death in men in most countries [2]. Incidence rates varied sixfold between countries and varied between registries within some countries, such as Brazil (threefold–sixfold). Mortality rates varied fourfold between countries. Reasons for geographical and temporal variation in prostate cancer incidence and mortality rates include differences in diagnostic and registration practices, healthcare access, treatment and death certification, and public awareness. By 2030, the burden from prostate cancer is expected to nearly double in this region, mainly due to the simultaneous expansion and age distribution of the male population.

For appropriate patients, androgen deprivation therapy (ADT) may be achieved through either surgical or medical castration. Unfortunately, despite ADT, disease will invariably progress to castration-resistant prostate cancer (CRPC). Approximately 90% of patients with CRPC develop bone metastases (mCRPC), which may account for significant morbidity and mortality associated with the disease [3, 4].

In 2004, docetaxel (taxane chemotherapy) was the first survival-prolonging treatment to be approved for mCRPC [5, 6]. Since then, five new agents have been approved in this setting including sipuleucel-T (immunotherapy, only in the United States, [US]) [7], cabazitaxel (taxane chemotherapy) [8], abiraterone [9–12] and enzalutamide (androgen receptor [AR] axis-targeted therapies) [13, 14], and radium-223 (targeted alpha therapy) [15]. All of these agents have significantly improved overall survival for patients with mCRPC, with median survival times of 35 months reported in chemonaive patients treated with AR axis-targeted agents (supplementary table S1). Despite regulatory approvals by the US Food and Drug Administration, European Medicines Agency or individual Latin American agencies, accessibility is not universal for all of these agents.

The strength of recommendation from treatment guidelines varies [16–19] (supplementary table S2). The Prostate Cancer Clinical Trials Working Group (PCWG) provides recommendations in relation to trial design in this setting [20], and consensus recommendations from experts treating patients in current clinical practice are available [21–24]. However, level 1 data for optimal patient selection, treatment combinations or sequencing are limited. These choices are currently influenced by clinical judgment, therapeutic experience and availability. Access to life-extending yet costly new therapies varies globally, with limited public reimbursement being of particular concern to patients and to physicians treating mCRPC in Latin America.

The Latin American mCRPC treatment committee, comprising a multidisciplinary group of 14 uro-oncology experts from Argentina, Brazil, Colombia, Mexico and the USA, was convened in 2016 in Bogotá, Colombia. These Latin American counties were selected as they currently have the highest adoption of the approved life-prolonging treatments for mCRPC. In addition, clinical expertise from these countries has contributed to developing current treatment guidelines (e.g., Brazil and Argentina for NCCN guidelines). A representative from the USA, experienced in the clinical development of treatment therapies and guidelines for mCRPC, was included to moderate discussion. The expert committee reviewed the current evidence and guidance for treating mCRPC, considering factors influencing patient selection, the timing of treatment and best methods for treatment monitoring. Participants provided their clinical opinion and experience that might inform such decisions to develop consensus recommendations that may assist other Latin American physicians in clinical decision making (Table 1).

**Table 1:** Summary of treatment recommendations for approved agents in mCRPC in Latin America

Agent	Initiation	Switch/stop	Monitoring treatment
Chemotherapy			
Docetaxel^a^	Patients with rapidly progressive (symptomatic) and/or high burden of disease	Clinical indication/radiographic progression	Radiographic methodology depends on availability. Bone and CT scans are standard imaging techniques for diagnosing new bone lesions. CT scan is useful for monitoring extra-skeletal progression whilst on treatment
Cabazitaxel	Patients progressing (who do not respond or have a short response) on docetaxel		
AR axis therapy^b^			
Abiraterone acetate	First-line for mildly symptomatic/asymptomatic patients Patients progressing on chemotherapy Patients progressing on radium-223 if a AR axis-targeted therapy has not been previously used	Clinical indication/radiographic progression Switching from abiraterone to enzalutamide and vice versa is not recommended	MRI and PET with new molecular tracers may prove useful in the future for monitoring disease progression but require validation. There are currently no new validated biomarkers for monitoring and treatment decisions. Clinical biomarker studies are ongoing. PSA values alone should be interpreted with caution. Biomarker information should be used with patient clinical parameters
Enzalutamide	As above Preference for enzalutamide for patients with visceral metastases		
Targeted alpha therapy			
Radium-223^c^	In patients with symptomatic bone metastases Ideally in patients with bone metastases progressing on first-line abiraterone or enzalutamide (earlier in the course of mCRPC) Can be used prior to chemotherapy (patients unfit/unwilling for chemotherapy) Can be used in patients progressing on chemotherapy	Clinical indication/radiographic progression (evidence of visceral metastases) Patients with a rising PSA during radium-223 therapy and no visceral disease, adding a corticosteroid or an older hormone therapy are viable options	In the future, ALP may be validated as a more suitable marker than PSA for monitoring response to radium-223

*ALP* alkaline phosphatase, *CT* computed tomography, *mCRPC* metastatic castration-resistant prostate cancer, *MRI* magnetic resonance imaging, *PET* positron emission tomography, *PSA* prostate-specific antigen

^a^Docetaxel + androgen depravation therapy is also preferred for high-volume, metastatic hormone-sensitive disease

^b^Bicalutamide was recommended as a treatment alternative in cases where access to new hormone therapy is restricted or the aim is to expose patients to an additional line of therapy; however, results are expected to be worse than those with abiraterone or enzalutamide

^c^For the purpose of prolonging survival (not pain palliation) in patients with bone-predominant disease (no visceral metastases). Symptoms should be carefully assessed. Giving earlier in the course increases likelihood of receiving all six treatment cycles of life-prolonging therapy

## Methods

The multidisciplinary expert panel from Latin America reviewed the current recommendation for the use of chemotherapy (docetaxel and cabazitaxel), AR axis-targeted agents (abiraterone and enzalutamide) and targeted alpha therapy (radium-223) in the treatment of patients with mCRPC. Treatment guidelines reviewed were ESMO, NCCN, AUA and ASCO. Level 1 recommendations from these treatment guidelines were discussed in the context of the participants own clinical experience to develop clinical consensus, with consideration given to factors influencing patient selection and treatment monitoring. Voting was coordinated by Professor Sade; consensus recommendations were agreed on a majority vote. Dr Shore moderated discussion but did not contribute a vote.

### Current treatment options for mCRPC

Current treatment options for mCRPC are summarized (supplementary table S1). The integration of available agents for the optimal management of patients in Latin America was discussed.

### Chemotherapy

The TAX 327 [5] and SWOG 9916 [6] studies showed a survival benefit for patients with mCRPC treated with docetaxel-based chemotherapy compared with mitoxantrone-based control. The TROPIC study [8] demonstrated cabazitaxel (25 mg/m^2^) as a chemotherapeutic treatment option post-docetaxel. Subsequently, cabazitaxel 20 mg/m^2^ demonstrated non-inferiority for overall survival with an improved safety profile compared with 25 mg/m^2^ cabazitaxel in patients progressing on docetaxel [25]. However, both 20 and 25 mg/m^2^ cabazitaxel did not demonstrate superiority for survival compared with docetaxel in chemotherapy-naïve patients [26].

US and European guidelines recommend docetaxel for the treatment of patients with mCRPC and cabazitaxel in patients progressing on docetaxel [16, 18], and in some guidelines chemotherapy is recommended particularly in symptomatic patients, or in asymptomatic patients showing signs of rapid progression or visceral metastases [16]. There is consensus for taxane use (second-line) in patients with symptomatic disease progressing on AR axis-targeted agents, and many clinicians would use third-line cabazitaxel in patients progressing on AR axis-targeted agents (e.g., abiraterone and enzalutamide) and docetaxel [22]. Small retrospective studies suggest that prior treatment with AR axis-targeted agents may negatively impact on the activity of subsequent docetaxel treatment [27, 28].

The panel agreed that docetaxel should be recommended in patients with symptomatic mCRPC with rapidly progressive and/or high-burden disease. Cabazitaxel (20 mg/m^2^) was recommended for patients progressing on docetaxel.

### AR axis-targeted agents

The addition of the CYP17 inhibitor abiraterone to prednisone improved overall survival both in mCRPC patients progressing on docetaxel [9, 10], and first-line in asymptomatic or mildly symptomatic chemotherapy-naive patients (without visceral metastases) [11, 12].

In patients with mCRPC who had previously received chemotherapy, survival was longer in those treated with the AR inhibitor enzalutamide compared with placebo [13]. In chemotherapy-naïve patients, those who received enzalutamide showed benefit for radiological progression and overall survival compared with patients who received placebo [14]. Radiological progression was also improved in patients with visceral metastases.

Abiraterone and enzalutamide are approved for the first-line treatment of mCRPC, and in patients who have previously received docetaxel [16–19]. There was consensus for the first-line use of AR axis-targeted agents in patients with asymptomatic mCRPC [22]; treatment in patients with mCRPC leads to prostate-specific antigen (PSA) decline in 90% of cases. However after a PSA nadir, the majority of patients will experience a PSA rise. Switching from abiraterone to enzalutamide or vice versa on progression does not appear to be effective due to the development of resistance mechanisms [21, 29, 30].

The panel agreed that abiraterone and enzalutamide should be recommended as first-line treatment options for asymptomatic or mildly symptomatic patients with a rising PSA or low-volume mCRPC following ADT alone. Switching between the two agents for second-line treatment was not recommended. Enzalutamide was recommended as the hormone therapy of choice for patients with visceral metastases. Both agents were considered appropriate options for patients progressing on chemotherapy.

In some Latin American countries, the use of bicalutamide is considered as a viable first-line option for chemotherapy-naive patients progressing slowly after ADT alone, particularly for those with low burden of disease and slowly rising PSA. However, abiraterone or enzalutamide is considered to be more active than bicalutamide in this setting and should be preferred, if available, considering their survival-prolonging effects [31, 32].

The panel agreed to recommend bicalutamide as a treatment alternative in cases where access to abiraterone or enzalutamide is restricted, or where the aim is to expose patients to an additional line of therapy.

### Targeted alpha therapy

Radium-223 is incorporated into new bone in areas of increased bone turnover around prostate cancer metastases [33]. Radium-223 emits high-energy alpha particles over a short range (< 0.1 mm) resulting in a localized antitumor effect and minimal toxicity to normal bone marrow [33–35].

In the phase 3 ALSYMPCA study in patients with mCRPC who had ≥ 2 symptomatic bone metastases (no visceral or bulky nodal disease), overall survival was longer, time to first on-treatment symptomatic skeletal event (SSE) was delayed, and a meaningful improvement in patient quality of life (QoL) was reported, in those receiving radium-223 compared with placebo [15]. Patients in both arms also received best standard of care (BSoC). Radium-223 is recommended for patients with symptomatic bone-predominant mCRPC without visceral metastases [16–19]. Whilst radium-223 reduced pain in this study, this was not a primary measure of treatment response and was not observed in all patients. Failure to reduce pain should not lead to discontinuation of radium-223. Administering analgesics or external beam radiation therapy should be considered, and radium-223 can be safely and effectively combined with these and other agents in BSoC [15, 36, 37].

The panel recommend that radium-223 should be administered primarily for the purpose of prolonging overall survival and not for pain palliation.

Radium-223 is considered easy to administer with a good safety profile [15]. Administration should be performed by physicians and health technicians certified to use radioisotopes, with appropriate radiation safety precautions taken. The importance of educating both patients and staff on radium-223 use is emphasized; it is safe for appropriately trained healthcare workers [38].

The timing of the administration of radium-223 prior to the onset of visceral disease is important. Approximately 90% of patients with a life expectancy of > 1 year will have bone metastases and no visceral disease [39] and are potential candidates for radium-223. In the USA and Europe, some clinicians suggest that radium-223 may be given following early signs of progression after first-line abiraterone or enzalutamide [22, 23]. Exploratory analyses suggest that patients with less advanced mCRPC are more likely to complete the full six cycles of radium-223 [40, 41]. Post hoc analyses reported the combination of radium-223 with abiraterone or enzalutamide was tolerable and may lead to longer survival [37, 42]. Phase 3 clinical trials including ERA-223 (NCT02043678) and PEACE-3 (NCT02194842) in asymptomatic/mildly symptomatic patients should provide level 1 evidence for the combination of radium-223 with AR axis-targeted agents.

Data from the phase 3 study showed radium-223 was effective when administered to patients with or without previous docetaxel use [43]. In further exploratory analyses in patients who received docetaxel after radium-223, it was shown that docetaxel was feasible and well tolerated [44]. With the potential for additive myelosuppression, radium-223 is not recommended for use in combination with chemotherapy outside of a clinical trial [45].

There was consensus that the number of bone metastases should not influence decisions on whether to administer radium-223. In the absence of level 1 evidence, for guidance, use of radium-223 early in the disease course in patients with CRPC and bone metastases (no visceral disease) progressing on first-line abiraterone or enzalutamide may be considered. Radium-223 can also be used for patients progressing on chemotherapy (with no visceral metastases), but should not be used in combination with chemotherapy. Chemotherapy can be safely administered after radium-223.

In instances when PSA is rising during radium-223 therapy, and in the absence of radiological progression, or clinical indication (no visceral metastases), some physicians suggest the addition of abiraterone or enzalutamide. Due to lack of reimbursement for this combination in Latin America, adding a corticosteroid (e.g., low-dose dexamethasone) here would be an option with a view to starting abiraterone or enzalutamide after six cycles of radium-223 therapy. An alternative option would be to add an older antihormone therapy (bicalutamide, diethylstilbestrol and ketoconazole) in line with BSoC use in the radium-223 phase 3 study [15]. This approach is used in some countries including Mexico and Argentina.

The panel agreed that for patients with a rising PSA during radium-223 therapy and no visceral disease, adding a corticosteroid or an older anti-hormone therapy are viable options. If the patient has not received abiraterone or enzalutamide, starting either of those after radium-223 (6 cycles) is also an option.

### Patient selection and treatment monitoring

Optimal disease management relies on selecting patients who will benefit most from a particular agent, and knowing when best to change treatment. The PCWG3 have introduced the concept of ‘no longer clinically benefiting’ to underscore the distinction between first evidence of progression and the clinical need to terminate treatment in clinical trials [20]. In addition, two out of three criteria consisting of PSA progression, radiographic progression and clinical deterioration are recommended requirements in order to stop treatment [21]. In this context, factors important for patient selection and treatment monitoring were discussed.

### Patient symptoms

Disease symptoms are important considerations in treatment selection [15, 22], although accurate symptom evaluation is problematic. The symptom definition used in the ALSYMPCA study is regarded as standard for symptomatic patient identification (regular use of analgesic medication or treatment with EBRT required for cancer-related bone pain within the previous 12 weeks). However, patients treated with abiraterone or enzalutamide may complain of fatigue, muscle pain, sarcopenia amongst other symptoms, making it difficult to ascertain symptoms associated with metastatic bone disease, medication or other non-cancer-related conditions. Furthermore, reporting of pain varies widely according to patient age, cultural background and socioeconomic factors. Also patients taking over-the-counter drugs may consider themselves asymptomatic. Other important factors for consideration during symptom assessments include difficulty in sleeping, changes in daily activities, depression and weakness [46].

The panel recommend that in addition to assessing pain, clinicians should screen for the presence of other symptoms including depression, sarcopenia, weakness and changes to daily life. It was advised that including a caregiver (family member, close friend or professional caregiver) during the consultation process is very useful for the evaluation of patient symptoms [46].

### Previous treatment history

Patient history of previous use of anticancer agents is important in treatment selection. This information in the hormone-sensitive setting may be important when considering treatment in patients who have progressed to mCRPC. The STAMPEDE [47] and CHAARTED [48] trials support the combination of chemotherapy with ADT in newly diagnosed patients with high-volume metastatic hormone-sensitive disease, which is now considered to be the standard of care for these patients. However, chemotherapy use in metastatic hormone-sensitive disease varies worldwide. In the USA, chemotherapy with ADT is predominantly given to patients with high-volume metastatic disease [48] although in the UK it is also provided to patients with low-volume disease [47]. Some variation is found across countries in Latin America, but predominantly chemotherapy is combined with ADT in patients with high-volume disease. In these cases, many clinicians will typically wait 1–2 months after the start of ADT before administering docetaxel. Approaches will vary depending on whether physicians are working in university- or community-based centers.

The panel agreed that the combined use of docetaxel and ADT in high-volume, metastatic, hormone-sensitive disease is recommended.

Recent data have demonstrated that adding abiraterone to ADT in patients with newly diagnosed high-risk metastatic ‘hormone-naïve’ prostate cancer significantly prolonged survival [49, 50]. Thus, where access is available in Latin American countries, the use of abiraterone in the hormone-sensitive setting may also influence the subsequent choice of agents in patients progressing to mCRPC.

### Biomarkers

Currently, there are no validated biomarkers for predicting which patients might benefit from a specific treatment. Recent data suggest that screening for AR gene alterations may identify patients unlikely to respond to AR axis-targeted therapies. Indeed, the presence of AR splice variant-7 (AR-V7) mRNA in circulating tumor cells from patients with mCRPC was associated with resistance to enzalutamide and abiraterone [51]. Furthermore, a gain in tumor AR copy number was associated with worse patient outcome following treatment with enzalutamide or abiraterone [52]. However, such potential diagnostic approaches require further clinical validation before their routine implementation in the community setting/clinical practice. Clinical studies investigating biomarkers in mCRPC (AR-V7) are ongoing (e.g., NCT03103724 and NCT02429193). Recently, data from several clinical trials have been shared in the DREAM (Dialogue for Reverse Engineering Assessments and Methods) challenge, which reported novel prognostic models (based on patient demographics, laboratory values, medical history, lesion sites and previous treatments) for predicting overall survival in patients with mCRPC [53]. Such approaches may be useful for identifying predictive markers for response to treatment in this setting.

Rising PSA levels and short PSA doubling times during treatment may indicate tumor progression. However, in clinical practice, PSA progression alone requires cautious interpretation and is not recommended as a trigger to stop or switch a treatment [21, 22]. In particular, a PSA flare phenomenon has been described following the initiation (first 2–3 months) of chemotherapy and treatment with AR axis-targeted agents. Rapidly rising serum ALP levels (a marker of osteoblast activity) is an indication of disease progression in bone. Serum ALP levels may therefore be a more clinically relevant biomarker than PSA in patients with bone metastases [21, 22]. Biomarker analysis from the ALSYMPCA study showed that radium-223 treatment led to a significant decrease in total (t)ALP levels compared with placebo, and patients receiving radium-223 who experienced a tALP decline at week 12 had longer overall survival than those who did not [15, 54]. By contrast, radium-223 had a relatively modest effect on PSA kinetics which was not a good surrogate for response [54].

The panel agreed that currently there are no validated biomarkers for predicting patients that might respond to therapy, and no new validated biomarkers for measuring on-treatment progression, although clinical biomarker studies are ongoing. Changes in PSA levels should be interpreted with caution and treatment should not be changed/stopped based on PSA alone. Imaging and clinical indicators of progression are also important. ALP may prove to be a more suitable marker of response to radium-223.

### Imaging

Guidelines for imaging metastatic disease are available [20, 55]. Imaging methods for patients with mCRPC and bone metastases are summarized (supplementary table S3).

Bone scintigraphy and computed tomography (CT) scans of the chest–thorax and abdomen–pelvis are recommended for patients with mCRPC before a new line of treatment is started [21, 23]. Data suggest that automated bone scan index (aBSI) at baseline is prognostic for survival, radiological progression-free survival and SSEs [56]. The use of magnetic resonance imaging (MRI), positron emission tomography (PET)/CT with labeled tracers (including prostate-specific membrane antigen [PMSA]) and single-photon emission CT bone scans (SPECT) combined with CT in the baseline staging and monitoring of treatment requires further investigation [16, 20, 21].

Access to different imaging methodologies varies across Latin America, and availability may be limited to academic centers. The standard imaging in Latin America for patients with rising PSA is a bone scan, and CT for the diagnosis of visceral metastases. However, where a PSA rise is measured, it was agreed that in the absence of clinical or radiographic progression, current treatment should continue.

The panel agree that bone scans and CT are the standard imaging tests in Latin America for detecting bone and visceral lesions. In patients with a rising PSA during ADT alone, and for patients with de novo metastatic disease, the panel recommends against imaging in the absence of laboratory abnormalities or patient complaints.

There is a lack of data on the use of imaging techniques for monitoring treatment response in clinical trials. Agreement on the frequency of use of imaging during treatment varies with some clinicians recommending imaging only when clinically indicated [21–23].

Monitoring of disease progression and treatment response in the bone is problematic, with bone lesion flare occurring on bone scans (scintigraphy using technetium-99 m-methyldiphosphate) [57, 58]. Whilst bone scans can detect new metastatic lesions, it is difficult to differentiate between tumor activity and the healing processes in the bone, making the distinction between spread of metastases and successful treatment difficult [58]. Frequent bone scintigraphy is therefore unlikely to be helpful. Further studies are necessary to confirm the role of systematic disease monitoring using more advanced techniques including MRI (whole body) or PSMA PET/CT in monitoring and characterizing early response to treatment [59]. The use of molecular imaging techniques including hyperpolarized carbon-13 MRI (NCT02844647), diffusion weighted MRI and PET/CT using novel tracers is under development (supplementary table S3).

For patients with mCRPC undergoing treatment with life-prolonging agents, the panel recommend that imaging should be prompted where symptomatic decline and/or significant worsening of laboratory evaluations are reported, or where a course of therapy has been successfully completed and another antineoplastic agent is under consideration. Where indicated, CT and bone scans are the standard imaging tests for patients with a rising PSA. Imaging should not be used to measure treatment response; a CT scan is recommended only if clinically indicated to check for signs of progression (visceral disease). Newly developed molecular imaging techniques may be useful in monitoring disease progression in bone and to detect extra-skeletal lesions, however these techniques require validation in prospective studies.

### Treatment cost and access to agents in Latin America

Unequal access to life-prolonging agents and to the newer technologies for monitoring treatment is a major limitation to successfully treating patients with mCRPC across different countries in Latin America. Treatment cost is a primary factor for limiting access to cancer agents. This may in part be due to the rise in the incidence of chronic diseases including cancer, which now compete for resources previously predominantly allocated to treating infectious diseases [60].

Obtaining adequate financing for cancer treatment is a challenge for many Latin American countries. The budget percentage spent for health in Latin America is generally lower than that of developed countries, although there are clear disparities between countries in Latin America; for example, Argentina spends 8.1% of its gross domestic product on health, whereas Brazil, the most populous country of the region, spends only 6.3% [60]. In the majority of these cases, however, health expenditures are concentrated in the private sector. For example in Mexico in 2008, of the total spent on health, 52% was for the private sector, which covers only 5% of the entire population [60].

Other factors influencing the optimal treatment of patients include: a fragmented structure and organization of healthcare systems in some countries, and unequal distribution of resources which are concentrated primarily in urban areas [60, 61]. In addition, in some regions, provision of education on the handling and administration of the use of radiotracers and alpha-targeted therapies may be required to improve knowledge of these agents.

With the treatment landscape for prostate cancer dramatically changing in the last decade, it is important to consider the added value regarding improved clinical outcome and QoL, and reduced morbidity to the patient, in addition to the cost of these new drugs. For example, continued use of older hormone therapies, whilst an option, may not be as effective as using newer AR axis-targeted agents. It is therefore important that there is dialogue between prostate cancer specialists and hospital and government decision makers, to educate on the clinical and economic value for preventing disease progression and the associated morbidities and complications (emergency room visits, hospital resource utilization), and to improve awareness of the importance of allocating funding in line with new evidenced-based (level 1) findings.

## Conclusions

Treatment options for patients with metastatic prostate cancer are increasing. Patients should have the opportunity to receive as many of the available life-prolonging therapies as possible. The recommendation for use of chemohormonal therapy for patients with metastatic hormone-sensitive prostate cancer may affect subsequent treatment selection and sequence in patients progressing to mCRPC. The thorough assessment of symptoms in patients with mCRPC prior to treatment was considered to be important and should not be limited to the reporting of pain alone. Taxane-based chemotherapy was recommended for patients with rapidly progressive (symptomatic) and/or high burden of disease. First-line treatment with AR axis-targeted therapies was recommended for patients with asymptomatic or mildly symptomatic disease and could also be considered for patients progressing on chemotherapy. It was recommended that radium-223 should be administered primarily for the purpose of prolonging survival, and not for pain palliation. Use of radium-223 early in the disease course in patients with CRPC and bone metastases (no visceral disease) who are progressing on first-line abiraterone or enzalutamide may be considered. PSA was not considered to be a reliable biomarker for monitoring response to radium-223. Biomarker information was considered to be best used in the context of other assessments to establish clinical progression. CT and bone scans were considered to be the current standard imaging tests for baseline staging and treatment monitoring (with respect to detecting new lesions) of patients with mCRPC in Latin America. Repeated radiological assessments were not recommended, and unless otherwise indicated by clinical assessment, imaging was recommended at the beginning and end of treatment to establish a baseline for further treatments.

## Electronic supplementary material

Below is the link to the electronic supplementary material.

Supplementary material 1 (DOCX 34 kb)

Supplementary material 2 (DOCX 32 kb)

Supplementary material 3 (DOCX 34 kb)
