# Supplementary material for: Optimizing the treatment of metastatic castration-resistant prostate cancer: a Latin America perspective
Source: Med Oncol. 2018 Mar 19;35(4):56. doi: 10.1007/s12032-018-1105-8 (PMC5859699; doi:10.1007/s12032-018-1105-8)
Supplement: Supplementary file 1 — Supplementary material 1 (DOCX 34 kb) [file 12032_2018_1105_MOESM1_ESM.docx]

**Supplementary table S1**

**Optimizing the treatment of metastatic castration-resistant prostate cancer: A Latin America perspective**

Juan Pablo Sade,^1^ Carlos Alberto Vargas Báez,^2^ Martin Greco,^3^ Carlos Humberto Martínez,^4^ Miguel Ángel Álvarez Avitia,^5^ Carlos Palazzo,^6^ Narciso Hernández Toriz,^7^ Patricia Isabel Bernal Trujillo,^8^ Diogo Assed Bastos,^9^ Fabio Augusto Schutz,^10^ Santiago Bella,^11^ Lucas Nogueira,^12^ Neal D Shore^13^

^1^Instituto Alexander Fleming Buenos Aires, Argentina; ^2^Universitario Fundacion Santa Fe de Bogota, Bogota, Colombia; ^3^Centro de Educación Médica e Investigaciones Clínicas, Buenos Aires, Argentina; ^4^Unidad de Cancerología, Departamento de Cirugía, División de Urología Hospital Pablo Tobón Uribe Medellín, Antioquia, Colombia; ^5^Instituto Nacional de Cancerologia, Mexico City, Mexico; ^6^Department of Uro-Oncology Instituto de Diagnóstico y Tratamiento Sagrada Familia, Tucumán, Argentina; ^7^Hospital de Oncología Centro Médico Nacional Siglo XXI, Mexico City, Mexico; ^8^Department of Nuclear Medicine, Fundación Santa Fe de Bogota, Bogota, Colombia; ^9^Hospital Sírio- Libanês and Uro-Oncology Department of the Instituto do Câncer do Estado de São Paulo (ICESP), São Paulo, Brazil; ^10^Hospital São José, São Paulo, Brazil; ^11^Universidad Católica de Córdoba and the Clínica Universitaria Reina Fabiola, Córdoba, Argentina; ^12^MD Hospital das Clínicas, Universidade Federal de Minas Gerais, Belo Horizonte, Brazil; ^13^Carolina Urologic Research Center, Myrtle Beach, SC, USA.

Corresponding author:

Neal D Shore, MD, FACS

Department of Urology

Carolina Urologic Research Center

823 82nd Parkway

Myrtle Beach

SC 29572.

Phone: + 1 843 449 1010

E-mail: NShore@gsuro.com

**Table S1.** Summary of approved agents in mCRPC

| **Agent** | **Mechanism of action** | **Study [citation]** | **Treatment** | **Overall survival*** | **Main Toxicities** |
| --- | --- | --- | --- | --- | --- |
| Chemotherapy |  |  |  |  |  |
| Docetaxel | Microtubule inhibitor | TAX 327 [1] | Docetaxel + prednisolone vs mitoxantrone + prednisolone | 18.9 vs 16.5, 0.76 (0.62–0.94) p=0.009 | Diarrhea, hematological (neutropenia), cardiac events and neuropathy |
|  |  | SWOG 9916 [2] | Docetaxel + estramustine vs  mitoxantrone + prednisone | 17.5 vs 15.6, 0.80 (0.67–0.97) p=0.02 |  |
| Cabazitaxel | Microtubule inhibitor | TROPIC [3] | Cabazitaxel + prednisolone vs mitoxantrone + prednisolone | 15.1 vs 12.7, 0.70 (0.59–0.83), p<0.0001 | Diarrhea, hematological (neutropenia), cardiac events |
| AR axis-targeted |  |  |  |  |  |
| Abiraterone acetate (AA) | CYP17 inhibitor | COU-AA-301 [4,5] | AA + prednisolone vs placebo + prednisolone | 15.8 vs 11.2, 0.74 (0.64–0.86), p<0.0001 | Increased incidence of mineralocorticoid-related adverse events, hepatotoxicity and cardiac disorders. |
|  |  | COU-AA-302 [6,7]^†^ | AA + prednisolone vs placebo + prednisolone | 34·7 vs 30.3, 0.81 (0·70–0·93), p=0.0033 |  |
|  |  |  |  |  |  |
| Enzalutamide | Androgen receptor inhibitor | AFFIRM [8] | Enzalutamide vs placebo | 18.4 vs 13.6, 0.63 (0.53–0.75), p<0.001 | Fatigue, hypertension, and seizures. |
|  |  | PREVAIL [9]^†^ | Enzalutamide vs placebo | 32.4 vs 30.2, 0.71, (0.60–0.84), p<0.001 |  |
| Targeted alpha therapy |  |  |  |  |  |
| Radium-223 (Ra-223) | Alpha-particle emitting radionuclide | ALSYMPCA [10] | Ra-223 + BSoC vs placebo + BSoC | 14.9 vs 11.3, 0.70, (0.58–0.82), p<0.001 | Low myelosuppression rates and a low incidence of grade 3/4 adverse events. |
| Immunotherapy |  |  |  |  |  |
| Sipuleucel-T | ACT using recombinant fusion protein PAP–GM-CSF | IMPACT [11] | Sipuleucel-T vs placebo | 25.8 vs. 21.7, 0.77 (0.61–0.97), p=0.02 | Chills, fever, and headache. |

*Overall survival data are medians in months, hazard ratio (95% CI), p-value; ^†^In chemotherapy naive patients. *ACT* autologous cell therapy, *BSoC* best standard of care, *PAP* prostatic acid phosphatase, *GM-CSF* granulocyte–macrophage colony stimulating factor, *mCRPC* metastatic castration-resistant prostate cancer

**References**

[1] Tannock IF, de Wit R, Berry WR, Horti J, Pluzanska A, Chi KN, Oudard S, Theodore C, James ND, Turesson I, Rosenthal MA, Eisenberger MA (2004) Docetaxel plus prednisone or mitoxantrone plus prednisone for advanced prostate cancer. N Engl J Med 351:1502-1512

[2] Petrylak DP, Tangen CM, Hussain MH, Lara PN, Jr., Jones JA, Taplin ME, Burch PA, Berry D, Moinpour C, Kohli M, Benson MC, Small EJ, Raghavan D, Crawford ED (2004) Docetaxel and estramustine compared with mitoxantrone and prednisone for advanced refractory prostate cancer. N Engl J Med 351:1513-1520

[3] de Bono JS, Oudard S, Ozguroglu M, Hansen S, Machiels JP, Kocak I, Gravis G, Bodrogi I, Mackenzie MJ, Shen L, Roessner M, Gupta S, Sartor AO (2010) Prednisone plus cabazitaxel or mitoxantrone for metastatic castration-resistant prostate cancer progressing after docetaxel treatment: a randomised open-label trial. Lancet 376:1147-1154

[4] de Bono JS, Logothetis CJ, Molina A, Fizazi K, North S, Chu L, Chi KN, Jones RJ, Goodman OB, Jr., Saad F, Staffurth JN, Mainwaring P, Harland S, Flaig TW, Hutson TE, Cheng T, Patterson H, Hainsworth JD, Ryan CJ, Sternberg CN, Ellard SL, Flechon A, Saleh M, Scholz M, Efstathiou E, Zivi A, Bianchini D, Loriot Y, Chieffo N, Kheoh T, Haqq CM, Scher HI (2011) Abiraterone and increased survival in metastatic prostate cancer. N Engl J Med 364:1995-2005

[5] Fizazi K, Scher HI, Molina A, Logothetis CJ, Chi KN, Jones RJ, Staffurth JN, North S, Vogelzang NJ, Saad F, Mainwaring P, Harland S, Goodman OB, Jr., Sternberg CN, Li JH, Kheoh T, Haqq CM, de Bono JS (2012) Abiraterone acetate for treatment of metastatic castration-resistant prostate cancer: final overall survival analysis of the COU-AA-301 randomised, double-blind, placebo-controlled phase 3 study. Lancet Oncol 13:983-992

[6] Ryan CJ, Smith MR, de Bono JS, Molina A, Logothetis CJ, de Souza P, Fizazi K, Mainwaring P, Piulats JM, Ng S, Carles J, Mulders PF, Basch E, Small EJ, Saad F, Schrijvers D, Van Poppel H, Mukherjee SD, Suttmann H, Gerritsen WR, Flaig TW, George DJ, Yu EY, Efstathiou E, Pantuck A, Winquist E, Higano CS, Taplin ME, Park Y, Kheoh T, Griffin T, Scher HI, Rathkopf DE (2013) Abiraterone in metastatic prostate cancer without previous chemotherapy. N Engl J Med 368:138-148

[7] Ryan CJ, Smith MR, Fizazi K, Saad F, Mulders PF, Sternberg CN, Miller K, Logothetis CJ, Shore ND, Small EJ, Carles J, Flaig TW, Taplin ME, Higano CS, de Souza P, de Bono JS, Griffin TW, De Porre P, Yu MK, Park YC, Li J, Kheoh T, Naini V, Molina A, Rathkopf DE (2015) Abiraterone acetate plus prednisone versus placebo plus prednisone in chemotherapy-naive men with metastatic castration-resistant prostate cancer (COU-AA-302): final overall survival analysis of a randomised, double-blind, placebo-controlled phase 3 study. Lancet Oncol 16:152-160

[8] Scher HI, Fizazi K, Saad F, Taplin ME, Sternberg CN, Miller K, de Wit R, Mulders P, Chi KN, Shore ND, Armstrong AJ, Flaig TW, Flechon A, Mainwaring P, Fleming M, Hainsworth JD, Hirmand M, Selby B, Seely L, de Bono JS (2012) Increased survival with enzalutamide in prostate cancer after chemotherapy. N Engl J Med 367:1187-1197

[9] Beer TM, Armstrong AJ, Rathkopf DE, Loriot Y, Sternberg CN, Higano CS, Iversen P, Bhattacharya S, Carles J, Chowdhury S, Davis ID, de Bono JS, Evans CP, Fizazi K, Joshua AM, Kim CS, Kimura G, Mainwaring P, Mansbach H, Miller K, Noonberg SB, Perabo F, Phung D, Saad F, Scher HI, Taplin ME, Venner PM, Tombal B (2014) Enzalutamide in metastatic prostate cancer before chemotherapy. N Engl J Med

[10] Parker C, Nilsson S, Heinrich D, Helle SI, O'Sullivan JM, Fossa SD, Chodacki A, Wiechno P, Logue J, Seke M, Widmark A, Johannessen DC, Hoskin P, Bottomley D, James ND, Solberg A, Syndikus I, Kliment J, Wedel S, Boehmer S, Dall'Oglio M, Franzen L, Coleman R, Vogelzang NJ, O'Bryan-Tear CG, Staudacher K, Garcia-Vargas J, Shan M, Bruland OS, Sartor O (2013) Alpha emitter radium-223 and survival in metastatic prostate cancer. N Engl J Med 369:213-223

[11] Kantoff PW, Higano CS, Shore ND, Berger ER, Small EJ, Penson DF, Redfern CH, Ferrari AC, Dreicer R, Sims RB, Xu Y, Frohlich MW, Schellhammer PF (2010) Sipuleucel-T immunotherapy for castration-resistant prostate cancer. N Engl J Med 363:411-422

[
