# Supplementary material for: Optimizing the treatment of metastatic castration-resistant prostate cancer: a Latin America perspective
Source: Med Oncol. 2018 Mar 19;35(4):56. doi: 10.1007/s12032-018-1105-8 (PMC5859699; doi:10.1007/s12032-018-1105-8)
Supplement: Supplementary file 2 — Supplementary material 2 (DOCX 32 kb) [file 12032_2018_1105_MOESM2_ESM.docx]

**Supplementary table S2**

**Optimizing the treatment of metastatic castration-resistant prostate cancer: A Latin America perspective**

Juan Pablo Sade,^1^ Carlos Alberto Vargas Báez,^2^ Martin Greco,^3^ Carlos Humberto Martínez,^4^ Miguel Ángel Álvarez Avitia,^5^ Carlos Palazzo,^6^ Narciso Hernández Toriz,^7^ Patricia Isabel Bernal Trujillo,^8^ Diogo Assed Bastos,^9^ Fabio Augusto Schutz,^10^ Santiago Bella,^11^ Lucas Nogueira,^12^ Neal D Shore^13^

^1^Instituto Alexander Fleming Buenos Aires, Argentina; ^2^Universitario Fundacion Santa Fe de Bogota, Bogota, Colombia; ^3^Centro de Educación Médica e Investigaciones Clínicas, Buenos Aires, Argentina; ^4^Unidad de Cancerología, Departamento de Cirugía, División de Urología Hospital Pablo Tobón Uribe Medellín, Antioquia, Colombia; ^5^Instituto Nacional de Cancerologia, Mexico City, Mexico; ^6^Department of Uro-Oncology Instituto de Diagnóstico y Tratamiento Sagrada Familia, Tucumán, Argentina; ^7^Hospital de Oncología Centro Médico Nacional Siglo XXI, Mexico City, Mexico; ^8^Department of Nuclear Medicine, Fundación Santa Fe de Bogota, Bogota, Colombia; ^9^Hospital Sírio- Libanês and Uro-Oncology Department of the Instituto do Câncer do Estado de São Paulo (ICESP), São Paulo, Brazil; ^10^Hospital São José, São Paulo, Brazil; ^11^Universidad Católica de Córdoba and the Clínica Universitaria Reina Fabiola, Córdoba, Argentina; ^12^MD Hospital das Clínicas, Universidade Federal de Minas Gerais, Belo Horizonte, Brazil; ^13^Carolina Urologic Research Center, Myrtle Beach, SC, USA.

Corresponding author:

Neal D Shore, MD, FACS

Department of Urology

Carolina Urologic Research Center

823 82nd Parkway

Myrtle Beach

SC 29572.

Phone: + 1 843 449 1010

E-mail: NShore@gsuro.com

**Table S2.** Summary of US and European treatment recommendations for approved agents in mCRPC

| **Agent** | **ASCO* (2014) [1]** | **AUA (2015) ^†^ [2]** | **NCCN (2017) ^‡^ [3]** | **ESMO (2015)^§^ [4]** | **EAU (2016)^¶^ [5]** |
| --- | --- | --- | --- | --- | --- |
| Docetaxel | Should be offered to patients with mCRPC | Index patient 2–5 | mCRPC:  primarily symptomatic or in asymptomatic patients showing signs of rapid progression or visceral metastases | mCRPC | Treat patients with mCRPC with life prolonging agents. Base the choice of first-line treatment on the performance status, symptoms, comorbidities and extent of disease (alphabetical order: abiraterone, docetaxel, enzalutamide, radium-223, sipuleucel-T)  Offer patients with mCRPC who are candidates for cytotoxic therapy docetaxel  In patients with mCRPC and progression following docetaxel chemotherapy, offer further life-prolonging treatment options, which include cabazitaxel, abiraterone,  enzalutamide and radium-223 |
| Cabazitaxel | Patients with mCRPC patients progressing on docetaxel (moderate recommendation) | Index patient 5 | mCRPC post docetaxel | mCRPC post docetaxel |  |
| Abiraterone acetate plus prednisolone | Should be offered to patients with mCRPC | Index patient 2–6 | Chemo naive, symptomatic/mildly symptomatic mCRPC  Chemo naive with visceral metastases (category 2A)^a^  mCRPC post docetaxel | Chemo naive, symptomatic/mildly symptomatic mCRPC  mCRPC post docetaxel |  |
| Enzalutamide | Should be offered to patients with mCRPC | Index patient 2­–6 | Chemo naive, symptomatic/mildly symptomatic mCRPC and or with visceral metastases  mCRPC post docetaxel | Chemo naive, symptomatic/mildly symptomatic mCRPC  mCRPC post docetaxel |  |
| Radium-223 | Should be offered to patients with mCRPC and bone metastases | Index patient 3–5 | mCRPC with symptomatic bone metastases and no visceral disease | Bone predominant symptomatic mCRPC with no visceral disease |  |
| Sipuleucel-T | Asymptomatic/minimally symptomatic mCRPC | Index patient 2 | Asymptomatic/minimally symptomatic mCRPC | - |  |

*Strong recommendation unless indicated. **^†^**Index patient: 1=asymptomatic non-metastatic CRPC; 2=asymptomatic or minimally-symptomatic, mCRPC without prior docetaxel chemotherapy; 3=symptomatic, mCRPC with good performance status and no prior docetaxel chemotherapy; 4=Symptomatic, mCRPC with poor performance status and no prior docetaxel chemotherapy; 5=Symptomatic, mCRPC with good performance status and prior docetaxel chemotherapy; 6=symptomatic, mCRPC with poor performance status and prior docetaxel chemotherapy. **^‡^**Data are NCCN category 1 recommendation unless indicated; ^a^For chemo naive mCRPC patients with visceral metastases who aren’t suitable for docetaxel-based therapy. ^§^Data are for recommendations with IA level evidence. ^¶^Grade A evidence shown unless otherwise stated. *mCRPC* metastatic castration-resistant prostate cancer.

**References**

[1] Basch E, Loblaw DA, Oliver TK, Carducci M, Chen RC, Frame JN, Garrels K, Hotte S, Kattan MW, Raghavan D, Saad F, Taplin ME, Walker-Dilks C, Williams J, Winquist E, Bennett CL, Wootton T, Rumble RB, Dusetzina SB, Virgo KS (2014) Systemic therapy in men with metastatic castration-resistant prostate cancer:American Society of Clinical Oncology and Cancer Care Ontario clinical practice guideline. J Clin Oncol 32:3436-3448

[2] Cookson MS, Lowrance WT, Murad MH, Kibel AS (2015) Castration-resistant prostate cancer: AUA guideline amendment. J Urol 193:491-499

[3] NCCN guidelines-Prostate cancer version 2.2017 https://www.nccn.org.

[4] Parker C, Gillessen S, Heidenreich A, Horwich A (2015) Cancer of the prostate: ESMO Clinical Practice Guidelines for diagnosis, treatment and follow-up. Ann Oncol 26 Suppl 5:v69-77

[5] Mottet N, Bellmunt J, Briers E, Bolla M, Cornford P, De Santis M, Henry A, Joniau S, Lam T, Mason MD, Matveev V, van der Poel H, van der Kwast TH, Rouvière O, Wiegel T, van den Bergh R, van den Broeck T, van Casteren NJ, Everaerts W, Marconi L, Moldovan P EAU-ESTRO-SIOG Guidelines on Prostate Cancer available at https://uroweb.org/wp-content/uploads/EAU-Guidelines-Prostate-Cancer-2016.pdf

.
