# Supplementary material for: Optimizing the treatment of metastatic castration-resistant prostate cancer: a Latin America perspective
Source: Med Oncol. 2018 Mar 19;35(4):56. doi: 10.1007/s12032-018-1105-8 (PMC5859699; doi:10.1007/s12032-018-1105-8)
Supplement: Supplementary file 3 — Supplementary material 3 (DOCX 34 kb) [file 12032_2018_1105_MOESM3_ESM.docx]

**Supplementary table S3**

**Optimizing the treatment of metastatic castration-resistant prostate cancer: A Latin America perspective**

Juan Pablo Sade,^1^ Carlos Alberto Vargas Báez,^2^ Martin Greco,^3^ Carlos Humberto Martínez,^4^ Miguel Ángel Álvarez Avitia,^5^ Carlos Palazzo,^6^ Narciso Hernández Toriz,^7^ Patricia Isabel Bernal Trujillo,^8^ Diogo Assed Bastos,^9^ Fabio Augusto Schutz,^10^ Santiago Bella,^11^ Lucas Nogueira,^12^ Neal D Shore^13^

^1^Instituto Alexander Fleming Buenos Aires, Argentina; ^2^Universitario Fundacion Santa Fe de Bogota, Bogota, Colombia; ^3^Centro de Educación Médica e Investigaciones Clínicas, Buenos Aires, Argentina; ^4^Unidad de Cancerología, Departamento de Cirugía, División de Urología Hospital Pablo Tobón Uribe Medellín, Antioquia, Colombia; ^5^Instituto Nacional de Cancerologia, Mexico City, Mexico; ^6^Department of Uro-Oncology Instituto de Diagnóstico y Tratamiento Sagrada Familia, Tucumán, Argentina; ^7^Hospital de Oncología Centro Médico Nacional Siglo XXI, Mexico City, Mexico; ^8^Department of Nuclear Medicine, Fundación Santa Fe de Bogota, Bogota, Colombia; ^9^Hospital Sírio- Libanês and Uro-Oncology Department of the Instituto do Câncer do Estado de São Paulo (ICESP), São Paulo, Brazil; ^10^Hospital São José, São Paulo, Brazil; ^11^Universidad Católica de Córdoba and the Clínica Universitaria Reina Fabiola, Córdoba, Argentina; ^12^MD Hospital das Clínicas, Universidade Federal de Minas Gerais, Belo Horizonte, Brazil; ^13^Carolina Urologic Research Center, Myrtle Beach, SC, USA.

Corresponding author:

Neal D Shore, MD, FACS

Department of Urology

Carolina Urologic Research Center

823 82nd Parkway

Myrtle Beach

SC 29572.

Phone: + 1 843 449 1010

E-mail: NShore@gsuro.com

**Table S3** Imaging techniques in mCRPC with bone predominant metastases

| **Technique** | **Comments** |
| --- | --- |
| ^99m^Tc bone scan; (technetium-99m-methyl diphosphate) | Standard for detecting areas of high bone turnover (may indicate metastases)  Low specificity/sensitivity  Negative scan not sufficient to exclude metastases [1,2]  Interpretations may be limited by flare phenomenon [1]  Not suited for response to therapy |
| CT | High level of anatomic detail [3]  Interpretations may be limited by flare phenomenon [4] |
| SPECT/CT | More sensitive than bone scans for detection of bone metastases [5] |
| MRI (axil, wb, DWI) | Greater sensitivity and specificity than BS  Often used to evaluate findings from BS  Potential for early detection of bone metastases [1,3]  Access to soft tissue and entire skeleton (wbMRI)  Practicality in comparison with standard techniques is under investigation [1]  Availability may be limited under restrictive resources  Use for monitoring on treatment requires validation from clinical studies |
| ^18^F- NaF PET | Greater sensitivity than BS and SPECT for detecting bone metastases [3  Quantitative [1]  Use for monitoring on treatment requires validation from clinical studies |
| PET/CT with new tracers (^11^C-Choline/ ^68^Ga-PMSA/18- Fluciclovine) | Accurate detection of recurrences [3,6, 7]  Concurrent bone and soft tissue screening  Validation required in prospective trials, current data are lacking for diagnosis and monitoring on treatment [3, 7] |

Shown are imaging techniques used and in development for the detection of metastases in mCRPC. *BS* bone scan (technetium-99m-methyldiphosphate), *CT* computerized tomography, *DWI*, Diffusion weighted imaging, *^18^F* ^18^Fluoride, *^68^Ga* ^68^Gallium, *mCRPC* metastatic castration-resistant prostate cancer, *MRI* magnetic resonance imaging, *NaF* sodium fluoride, *PET* positron emission tomography, *PMSA* prostate-specific membrane antigen, *SPECT* single-photon emission computerized tomography, *wb* whole body.

**References**

[1] Ulmert D, Solnes L, Thorek D. Contemporary approaches for imaging skeletal metastasis. Bone Res 2015; 3: 15024

[2] Fitzpatrick JM, Bellmunt J, Fizazi K, Heidenreich, A, Sternberg, CN, Tombal B, Alcaraz A, Bahl, A, Bracarda S, Di Lorenzo G, Efstathiou E, Finn SP, Fossa S, Gillessen S, Kellokumpu-Lehtinen PL, Lecouvet FE, Oudard S, de Reijke TM, Robson CN, De Santis M, Seruga B. de Wit R. Optimal management of metastatic castration-resistant prostate cancer: highlights from a European Expert Consensus Panel. Eur J Cancer 2014; 50: 1617-27

[3] NCCN guidelines-Prostate cancer version 2.2017 <https://www.nccn.org>

[4] Gillessen S, Omlin A, Attard G, de Bono JS, Efstathiou E, Fizazi K, Halabi S, Nelson PS, Sartor O, Smith MR, Soule HR, Akaza H, Beer TM, Beltran H, Chinnaiyan AM, Daugaard G, Davis ID, De Santis M, Drake CG, Eeles RA, Fanti S, Gleave ME, Heidenreich A, Hussain M, James ND, Lecouvet FE, Logothetis CJ, Mastris K, Nilsson S, Oh WK, Olmos D, Padhani AR, Parker C, Rubin MA, Schalken JA, Scher HI, Sella A, Shore ND, Small EJ, Sternberg CN, Suzuki H, Sweeney CJ, Tannock IF, Tombal B (2015) Management of patients with advanced prostate cancer: recommendations of the St Gallen Advanced Prostate Cancer Consensus Conference (APCCC) 2015. Ann Oncol 26:1589-1604

[5] Jambor I, Kuisma A, Ramadan S, et al. Prospective evaluation of planar bone scintigraphy, SPECT, SPECT/CT, 18F-NaF PET/CT and whole body 1.5T MRI, including DWI, for the detection of bone metastases in high risk breast and prostate cancer patients: SKELETA clinical trial. Acta Oncol 2016; 55: 59-67

[6] Leiblich A, Stevens D, Sooriakumaran P. The utility of molecular imaging in prostate cancer. Curr Urol Rep 2016; 17: 26

[7] Perera M, Papa N, Christidis D, Wetherell D, Hofman MS, Murphy DG, Bolton D, Lawrentschuk N. Sensitivity, specificity, and predictors of positive 68Ga-prostate-specific membrane antigen positron emission tomography in advanced prostate cancer: a systematic review and meta-analysis. Eur Urol 2016; 70: 926-937.
